# Modeling Elekta VersaHD using the Varian Eclipse treatment planning system for photon beams: A single‐institution experience

**DOI:** 10.1002/acm2.12709

**Published:** 2019-08-30

**Authors:** You Zhang, Anh H. Le, Zhen Tian, Zohaib Iqbal, Tsuicheng Chiu, Xuejun Gu, Andrei Pugachev, Robert Reynolds, Yang K. Park, Mu‐Han Lin, Strahinja Stojadinovic

**Affiliations:** ^1^ UT Southwestern Medical Center Dallas TX USA; ^2^ Roswell Park Cancer Institute Buffalo NK USA; ^3^ Winship Cancer Institute of Emory University Atlanta GA USA; ^4^ Oregon Health and Science University Portland OR USA

**Keywords:** Commissioning, Eclipse, Elekta, Treatment planning system, Varian, VersaHD

## Abstract

The aim of this study was to report a single‐institution experience and commissioning data for Elekta VersaHD linear accelerators (LINACs) for photon beams in the Eclipse treatment planning system (TPS). Two VersaHD LINACs equipped with 160‐leaf collimators were commissioned. For each energy, the percent‐depth‐dose (PDD) curves, beam profiles, output factors, leaf transmission factors and dosimetric leaf gaps (DLGs) were acquired in accordance with the AAPM task group reports No. 45 and No. 106 and the vendor‐supplied documents. The measured data were imported into Eclipse TPS to build a VersaHD beam model. The model was validated by creating treatment plans spanning over the full‐spectrum of treatment sites and techniques used in our clinic. The quality assurance measurements were performed using MatriXX, ionization chamber, and radiochromic film. The DLG values were iteratively adjusted to optimize the agreement between planned and measured doses. Mobius, an independent LINAC logfile‐based quality assurance tool, was also commissioned both for routine intensity‐modulated radiation therapy (IMRT) QA and as a secondary check for the Eclipse VersaHD model. The Eclipse‐generated VersaHD model was in excellent agreement with the measured PDD curves and beam profiles. The measured leaf transmission factors were less than 0.5% for all energies. The model validation study yielded absolute point dose agreement between ionization chamber measurements and Eclipse within ±4% for all cases. The comparison between Mobius and Eclipse, and between Mobius and ionization chamber measurements lead to absolute point dose agreement within ±5%. The corresponding 3D dose distributions evaluated with 3%global/2mm gamma criteria resulted in larger than 90% passing rates for all plans. The Eclipse TPS can model VersaHD LINACs with clinically acceptable accuracy. The model validation study and comparisons with Mobius demonstrated that the modeling of VersaHD in Eclipse necessitates further improvement to provide dosimetric accuracy on par with Varian LINACs.

## 1 INTRODUCTION

Varian Medical Systems and Elekta Instruments are two major vendors of medical linear accelerators (LINACs) for cancer radiation therapy. The latest generation of LINAC platforms, including TrueBeam/VitalBeam^1^ from Varian and VersaHD^2^ from Elekta, represent cutting edge technological advancements in radiotherapy treatment delivery. Both vendors also offer proprietary trademarked treatment planning systems: Varian Eclipse^3^ and Elekta Monaco^2^ for beam modeling, treatment dose calculation and plan design. In radiation oncology departments with a multivendor LINAC setting, utilization of more than one treatment planning system (TPS) creates another layer of complexity for data flow and systems communications, plan evaluations and comparisons, plan dose summations, and resource sharing. A single treatment planning system is the most cost‐effective and time‐efficient choice. However, challenges remain when a TPS from one vendor is used to model LINACs from another vendor due to general differences in LINAC architecture and proprietary solutions. Currently, there are very scarce resources such as publications or protocols available for guidance in this particular scenario. Another option is opting for a third party TPS from companies which specialize in modeling LINACs from any vendor, such as Pinnacle^4^ or Raystation.^5^ Clearly, LINAC vendors have an advantage in modeling their own LINACs due to patented and in‐depth internal knowledge needed for the highest dose calculation accuracy. In addition, integrated TPS and LINAC solutions in general enhance clinical workflow while programmatic vendor support can further increase efficiency.^6^ Moreover, a bundled purchase of LINAC and TPS from the same vendor may also allow substantial cost savings for the institution.

The recent acquisition of two VersaHD LINACs for our new departmental building coincided with the decision to switch to Eclipse TPS, which necessitated utilizing Eclipse to commission the Elekta LINACS. VersaHD LINACs feature a high resolution 160‐leaf Agility multileaf collimator (MLC) head^7^ with both flattened and flattening‐filter‐free (FFF) treatment beams.^8^ This article describes in detail the methodology of beam data acquisition, LINAC modeling, parameter optimization and dosimetric verification throughout the commissioning process for photon beams. A set of measurement‐based dose verification equipment (MatriXX,^9^ ionization chambers, and EBT3 radiochromic films) was combined with a computational and logfile‐based tool (Mobius^10^) to comprehensively evaluate the dosimetric accuracy of the LINAC treatment plans prior to clinical release. The goal of this work was to summarize the commissioning results for Elekta VersaHD LINACs within the Varian Eclipse TPS setting.

## 2 MATERIALS AND METHODS

### 2.1 Commissioning materials and equipment

At our institution, two Elekta VersaHD LINACs (Elekta Instruments AB, Stockholm, Sweden) were “matched” with each other and commissioned utilizing one mutual beam dataset for treatment planning and delivery. The VersaHD LINACs were accepted from Elekta with three flattened (6x, 10x and 15x) and two flattening‐filter‐free (6xFFF and 10xFFF) megavoltage photon energies. The available 6, 9, 12 and 15 megavoltage electron energies were accepted but not commissioned for clinical use. Each VersaHD LINAC is equipped with a 160‐leaf MLC set and a single pair of jaws in the other direction. The MLC consists of two opposed leaf banks with 80 leaves per bank. Each leaf has a projected width of 5 mm at the isocenter level. VersaHD is also equipped with a universal wedge^11^ which can be used with the 6x, 10x and 15x open fields. The Varian Eclipse (Varian Medical Systems, Palo Alto, CA) TPS version 13.6 was used to model the LINACs, and the Anisotropic Analytical Algorithm (AAA) was used for photon beam dose calculation.^12^

The Blue Phantom 2 water tank (IBA Dosimetry GmbH, Neu‐Isenburg, Germany) was used for beam scanning.^13^ The tank servo of 48 cm × 48 cm × 41 cm scanning volume was controlled by the OmniPro‐Accept 7 software (OmniPro‐Accept version 7.4, IBA Dosimetry GmbH, Neu‐Isenburg, Germany).^14^ The software provides automatic radiation detector navigation and data collection through a motion control unit. The software was also used for beam data postprocessing. All beam scanning and data collection were performed in agreement with manufacturer manuals and AAPM professional guidelines, including AAPM Task Group (TG) Reports No. 45^15^ and No. 106^16^. These materials provide detailed recommendations for acceptance testing and beam commissioning measurements, for both regular and small field sizes. The radiation detectors used with the corresponding detector properties and the associated tasks are listed in Table 1.

**Table 1:** Detectors used for VersaHD commissioning with detector properties and the corresponding tasks.

**Type**	Model	Sensitive volume	Diameter	Miscellaneous	Tasks performed
Cylindrical ionization chambers	Scanditronix CC13 (IBA Dosimetry GmbH, Neu‐Isenburg, Germany)	0.13 cm^3^	6 mm	C552 central electrode	PDD and profile (Photon FS ≥ 3 × 3 cm^2^)
	PTW Pinpoint 31014 (PTW, Freiburg, Germany)	0.015 cm^3^	2 mm	Aluminum central electrode	IMRT QA
	PTW Semiflex 31013 (PTW, Freiburg, Germany)	0.3 cm^3^	5.5 mm	Aluminum central electrode	Output factor (Photon FS ≥ 5 × 5 cm^2^) MLC transmission; Dosimetric leaf gap (DLG)
Diode field detectors	Sun Nuclear Edge (Sun Nuclear Corporation, Melbourne, FL)	0.0019 mm^3^	Active detection area: 0.8 × 0.8 mm^2^	Detecting at 0.3 mm geometric depth, or 0.5 mm water equivalent depth	PDD and profile (Photon FS ≤ 3 × 3 cm^2^); Output factor (Photon FS ≤ 5 × 5 cm^2^)

DLG, dosimetric leaf gaps; MLC, multileaf collimator; PDD, percent‐depth‐dose.

### 2.2 Beam data collection: PDDs and profiles

The PDDs and profiles were measured in accordance with the Varian Eclipse TPS reference manual for beam modelling. All measurements were performed at a 100 cm fixed source‐to‐surface distance (SSD). Field sizes ranged from 1 × 1 cm^2^ to 40 × 40 cm^2^ and were determined by jaws moving along radial axis direction and by MLCs moving along transverse direction. All fields used for scanning had two pairs of “guard leaves” abutting each other along the field edges under the diaphragm jaws. The other MLC leaves beyond the guard leaves, 1.0 cm from the field edge in the jaw direction, were closed by the LINAC per Elekta’s format. These field settings were created, stored as “Quick Beam” or “Stored Beam”, and used for beam scanning in the Elekta service mode. The cylindrical chamber position was automatically corrected for the effective point of measurement in the OmniPro‐Accept software. Beam scanning using the diode detector did not need the correction. All mandatory and recommended beam data measurements, such as PDD, crossline and inline profiles, were performed for desired field sizes and scanning depths (dmax, 5, 10, 20 and 30 cm), with and without the universal wedge. The diagonal profiles were measured for the maximum field size (40 × 40 cm^2^) at required scanning depths (dmax, 5, 10, 20 and 30 cm) with open beams. The central axis correction of the water phantom was performed for each beam energy setting prior to measurements. For field size (FS) ≤ 3 × 3 cm^2^, a Sun Nuclear Edge diode detector was used for its high spatial resolution and minimal susceptibility to the partial volume effect. The diode measurements were performed with no reference detector in the step‐by‐step scan mode as to increase the acquisition sampling time and improve the signal‐to‐noise ratio. In comparison, the beam data for FSs ≥ 3 × 3 cm^2^ were scanned continuously with a traditional dual ionization chamber setup, with field and reference Scanditronix CC13 cylindrical ionization chambers. As measurement conditions change, the scan speeds were adjusted to account for variations in dose rate, field size, and presence of beam modifiers (wedge) ensuring accurate and consistent quality measurements of the beam data. For 3 × 3 cm^2^ FS, both diode and cylindrical chamber measurements were performed for cross‐validation. For profile scans larger than the dimension of the water tank like 40 × 40 cm^2^ inline, crossline and diagonal profiles, half of the profile was acquired and the other half was mirrored. All PDDs and profiles were smoothed using a median filter with a sliding window size of 0.5 cm. The acquired PDDs and profiles of two VersaHD machines were matched within 1% for PDD and 1%/1 mm for beam profiles. The machines were adjusted accordingly until the deviations were smaller than these stated thresholds.

### 2.3 Beam data collection: output factors (Scp)

The output factors (Scp), as recommended by Varian, were measured at 95 cm SSD and 5 cm depth. The measurements were performed with the PTW Semiflex 31013 (PTW, Freiburg, Germany) cylindrical ionization chamber for field sizes ranging from 5 × 5 cm^2^ to maximum open field size of 40 × 40 cm^2^. The same measurements were also done for wedged fields up to the maximum wedged field size of 30 × 30 cm^2^. The data were normalized to the 10 × 10 cm^2^ field size reading. For smaller field sizes, FS ≤ 5 × 5 cm^2^, output factors were measured using the Edge diode detector. The corresponding normalized Scp factors were derived based on the “daisy chain” technique^14^ with the intermediate field size of 5 × 5 cm^2^. The universal wedge output factors were only measured for flattened beams. For wedged fields, the collimator was rotated by −90° to avoid detector placement in high dose gradient region along the wedge direction.

### 2.4 Leaf transmission and preliminary DLG measurements

Multileaf collimator transmission was measured separately for both MLC leaf banks. The leaves were parked to the rightmost position for one field, and then parked to the leftmost for the other, such that the field was blocked by one side’s leaf bank at each time. The commissioned value of MLC transmission was the average of both MLC banks. For all photon energies, the measurements were done utilizing a PTW Semiflex 31013 ionization chamber placed at a depth of 10 cm in a solid water phantom. The expected MLC transmission values should not exceed 2% according to the AAPM Task Group No. 50.^17^

Using the same setup, the DLG factor which accounts for transmission through leaf ends, was preliminarily measured for all photon energies using the sweeping‐gap technique^18^ by utilizing the corresponding Digital Imaging and Communications in Medicine (DICOM) plan files provided by Varian. For the sweeping gap technique, a series of MLC gaps of different sizes (6, 10, 14, 16, 20, 30, 40, and 50 mm) were individually swept from left to right throughout a rectangular field delivering a total of 500 MU. The MLC gap motion was set at a constant speed which resulted in a uniform fluence region of 120 mm × 200 mm. For each MLC gap the corresponding output reading was measured using the PTW Semiflex 31013 ionization chamber. The readings for different MLC gap sizes were affected by both gap and leaf transmission. After removing the contribution of MLC leaf transmission from the output reading, a linear fitting of corrected output readings (Y‐axis) as a function of MLC gaps (X‐axis) was performed. The x‐intercept of the fitted line, where the corrected output reading equaled zero, quantified the theoretical size of the MLC gap at which the field was “dosimetrically fully closed” (no output reading). Due to the rounded leaf‐end pattern and the resulting leaf‐end transmission, such an x‐intercept was a negative number, implying that MLC leaf pairs needed to be moved closer by an additional “x‐intercept” amount even if the LINAC showed they were “physically fully closed” (when MLC gap size equaled 0). Correspondingly, the absolute value of the “x‐intercept” indicated the DLG when the MLC leafs were physically closed.

### 2.5 Varian Eclipse TPS commissioning

#### 2.5.1 Beam modeling and DLG optimization using MatriXX

Next step involved importing measured beam data into Eclipse TPS for beam modeling. Consequently, a single model was built for two matched VersaHD LINACs. A set of commissioning plans was created for all energies using the initial DLG values from Section 2.D. The plans encompassed a full range of 2D/3D cases and intensity‐modulated radiation therapy (IMRT)/volumetric‐modulated arc therapy (VMAT) cases to cover our clinical practices. 2D/3D plans included three simple 4‐field box plans (field sizes ranging from 3 cm × 3 cm, 10 cm × 10 cm to 20 cm × 20 cm), a breast tangential plan using the field‐in‐field technique, a breast tangential plan using two wedged fields, a whole brain plan, a 3D lung SBRT plan using noncoplanar static fields, a 3D conformal‐arc spine plan, and a 3D pelvis plan using two wedged fields. All wedged plans used the Elekta universal wedges (motorized wedges) instead of the external hard wedges. All the plans, except for the wedged plans, were evaluated for all energies (6x/10x/15x/6xFFF/10xFFF). The wedged plans were evaluated for 6x/10x/15x only. Of note is that for the stated 2D/3D plans not all energies are suitable or clinically used, nevertheless, all were evaluated. IMRT/VMAT plans included noncoplanar brain IMRT, pelvis IMRT, head and neck VMAT, lung VMAT, lung IMRT, prostate VMAT, liver VMAT, and spine VMAT.

An iterative process for plan dose verification and DLG tuning was carried out for each energy. Note that the fine‐tuning of DLGs only involved the IMRT/VMAT plans, since the DLG values minimally affect dosimetry of basic 2D/3D plans. First, the plans were mapped to the MatriXX phantom (IBA Dosimetry, Bartlett, TN) and evaluated using MatriXX‐based QA protocol.^9^ The QA was performed using composite dose measurements with beam angles all fixed to 0 (a function provided by Eclipse when generating a verification plan). Second, based on the MatriXX results, the MLC DLG was adjusted in Eclipse. Third, the plans were re‐optimized and re‐calculated using the new MLC DLG value. The steps were iteratively repeated until the new DLG generated an average 90% or better gamma passing rate for all commissioning plans for each energy. The gamma method for MatriXX evaluation was based on the 2% global dose difference and 2 mm isodose distance criteria. The stricter 2%/2 mm criteria were used for MatriXX QA as measurements were performed with gantry angles all set to “0,” which reduced plan delivery uncertainties and in principle provided better dosimetric results.

#### 2.5.2 Film & ionization chamber plan verification and final DLG optimization

After the DLG optimization using MatriXX measurements, the commissioning plans were next mapped onto a solid water phantom. The solid water phantom, with a cross‐sectional area of 30 × 30 cm^2^ and a total thickness of 22 cm, was used for absolute point dose and 2D dose distribution measurements. The ionization chamber was centrally placed 11 cm below the anterior surface while the films were placed at 10 cm depth. Unlike the MatriXX QA, for which the beam delivery was simplified to fixed gantry (0°) and table (0°) angles, the film & ionization chamber QA used the original plan parameters including all beam angles, arc rotations and couch kicks intended to discover potential plan delivery issues, which represents the “true‐composite” scenario described in TG218. Film and ionization chamber measurements were used as the final dose verification and final DLG optimization step.

Point dose was measured using a PTW 31014 pinpoint chamber which was cross‐calibrated with an ADCL‐calibrated PTW Semiflex 31013 chamber. The PTW Semiflex 31013 Thimble chamber features a design similar to the classic Farmer chamber and offers equivalent accuracy for absolute dosimetry. Radiochromic EBT3 films (Ashland Advanced Materials, Bridgewater, NJ) were used for 2D absolute dose measurements and gamma analysis.^19^, ^20^ Films were scanned utilizing a flatbed scanner Epson 1000 XL (Epson America Inc., Long Beach, CA) 12 h after the exposure, consistent with the film dose response curve calibration process for absolute dosimetry. The OmniPro I’mRT software (IBA Dosimetry, Bartlett, TN) was used for film analysis. Taking into consideration the machine output variations, the absolute dose calibration uncertainty, the setup uncertainty, and the nonuniformity of the films, the passing criteria were set to ±5% for absolute point dose agreement for ionization chamber measurements and better than 90% gamma passing rate (3%global/2 mm) for EBT3 film absolute 2D dose distribution measurements. The selection of the ±5% threshold for the ionization chamber measurements accounts for the relatively large uncertainty of pinpoint chamber measurements^21^ and adheres to published IMRT QA action levels.^22^, ^23^ For gamma analysis, the global dose normalization with a 10% low‐dose threshold was used, in accordance with the recommendations of TG218. Based on the film and ionization chamber results, the DLG factors for all energies were further adjusted for clinical use.

#### 2.5.3 Commissioning logfile‐based Mobius as another plan and dose verification tool

A commercial DICOM‐RT‐based plan and delivery verification system Mobius (Mobius Medical Systems, Houston, TX, acquired by Varian recently) was commissioned to provide routine IMRT QA and to independently validate Eclipse model in addition to the ionization chamber and film measurements. The feasibility and accuracy of using Mobius for second dose check was reported in previous studies.^1^, ^2^ The commissioning was a similar iterative process involving Mobius DLG adjustments through plan recalculation for Mobius and Eclipse dose comparisons. The DLG factors in Mobius are not expected to be the same as DLG factors optimized for Eclipse as different beam models and dose calculation engines are employed for these two systems. Specifically, as a first step, the Mobius DLG values were optimized to create a “matched” plan dose distribution between Eclipse and Mobius for the commissioning plan cases described in Section 2.E.1. Similar to Eclipse, Mobius is utilizing DLG to model MLC edge leakage. It appears, however, that Mobius DLG values are intended to have broader impact by also allowing negative values which accounts for the dose calculation engine difference between Mobius and Eclipse in this case. The patient plans from Section 2.E.1 were initially used to set Mobius DLG values. In addition, the phantom‐mapped plans, Section 2.E.2, were imported into Mobius for dose calculation and to validate Mobius‐calculated ionization chamber doses relative to the ionization chamber measurements. Mobius DLGs values were then further adjusted until differences between the calculated and measured chamber doses were within ±5% for all commissioning plans.

## 3 RESULTS

### 3.1 Beam data measurements

**Figure 1:**
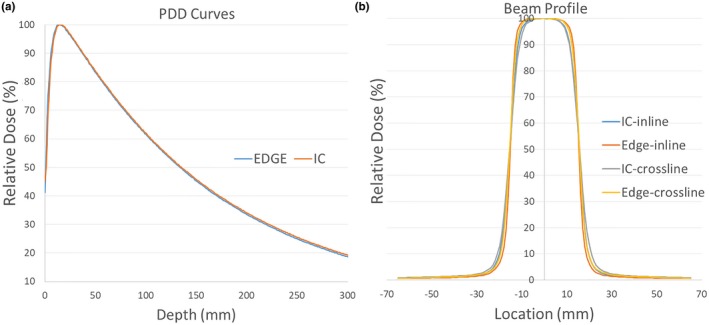
(a). 6x photon PDDs for 3 × 3 cm^2^ field size, red and blue curves represent ionization chamber and diode measurements, respectively. (b). Beam profiles for ionization chamber and diode measurements in inline and crossline planes. PDDs, percent‐depth‐dose.

**Figure 2:**
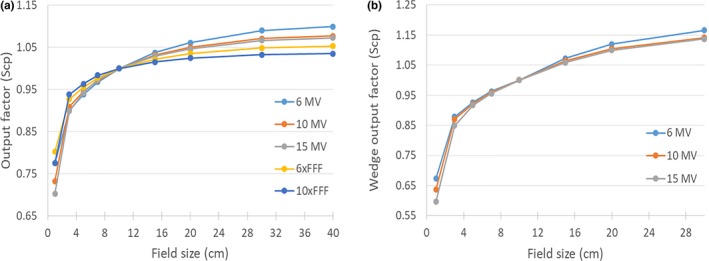
(a). Open field output factors measured for various field sizes and energies. (b). Wedge field output factors measured for various field sizes and energies.

All essential beam modeling dataset entries were measured and scrutinized before being imported into Eclipse. As an example, Fig. 1(a) shows measured PDD curves for 6x photons for a 3 cm × 3 cm field size. Red curve for ionization chamber (Scanditronix CC13, IC) and blue curve for diode (Sun Nuclear Edge, Edge) detector measurements reveal an excellent mutual agreement. The corresponding inline and crossline beam profiles at 1.5 cm depth are plotted in Fig. 1(b). The IC and Edge profiles matched very well except for small variations in sharp dose fall off regions. These variations are expected as the IC measurements include volume averaging effects proportional to IC's sensitive volume which is large relative to Edge detector. Figures 2(a) and 2(b) display open field and wedge field output factors which are in line with our institution’s prior data and in agreement with values from literature.^11^, ^24^

### 3.2 Beam modeling in Eclipse

**Figure 3:**
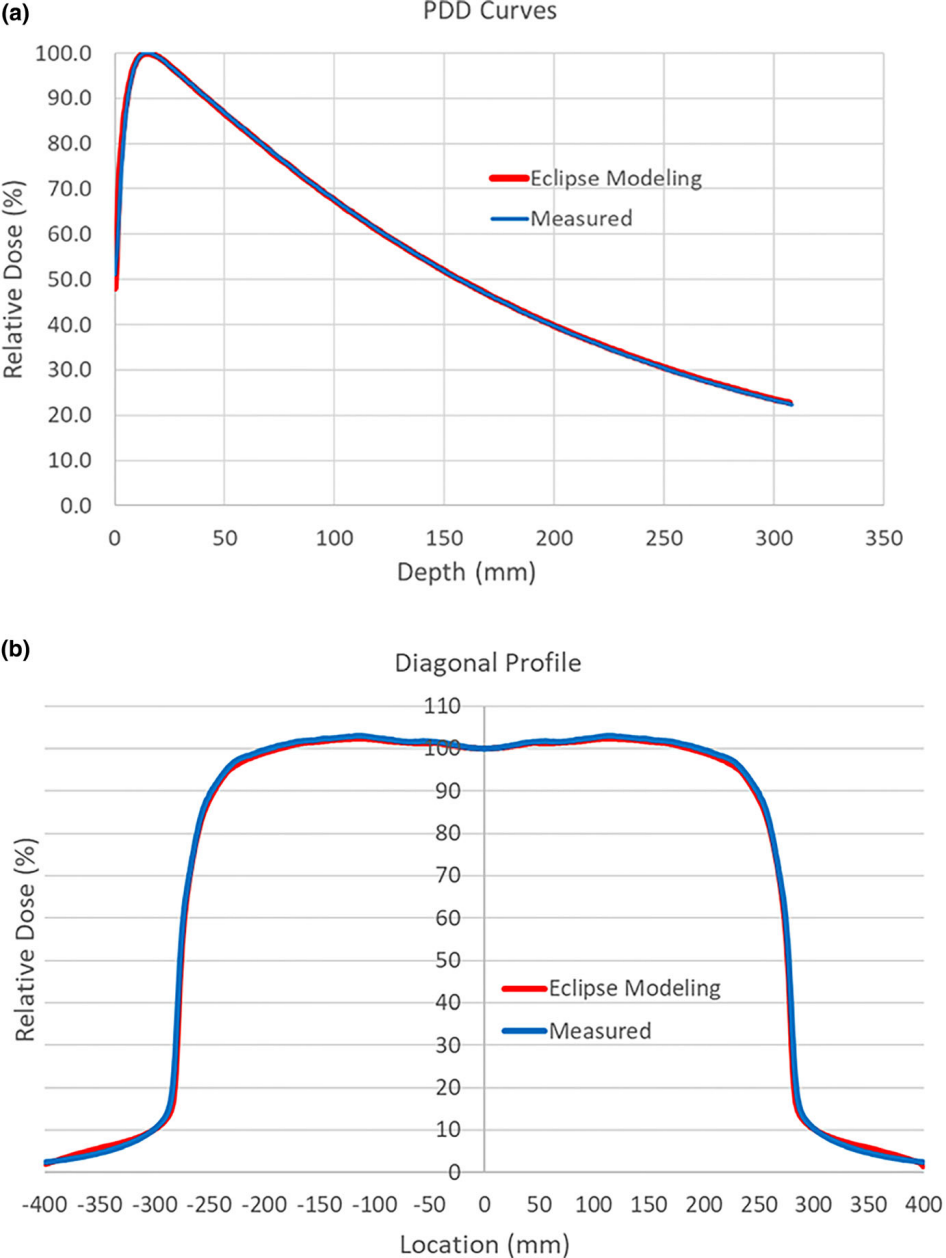
(a). Comparison between modeled and measured PDD curves for 6x beams at 10 cm × 10 cm field. (b). Comparison between modeled and measured diagonal profiles for 6× beams at 40 cm × 40 cm field, 10 cm depth.

The measured data were used as input in Eclipse for autofitting procedure which resulted in a VersaHD beam model. Fig. 3(a) shows measured and modeled 6x photons PDD curves for a 10 × 10 cm^2^ field size. Fig. 3(b) portrays measured and modeled 6x photons diagonal profiles for a 40 × 40 cm^2^ field size at 10 cm depth. There are slightly noticeable differences in the build‐up region for PDD curves and out of field variances for the profiles. Overall, the calculated and measured curves shown in red and blue colors are in essence superimposed and hard to distinguish one from the other indicating that the beams are correctly modeled. In general, the Eclipse‐modeled PDDs and profiles match the measurements within 1% deviation. Larger PDD and profile variations (>5%) can only be observed at the surface for PDDs, and around sharp fall‐off regions for profiles at shallow depths, respectively. The discrepancy is a combination of the modeling inaccuracy in build‐up regions and the LINAC intrinsic electron contamination, which is a universal problem to all TPS systems.

### 3.3 DLG measurements

**Figure 4:**
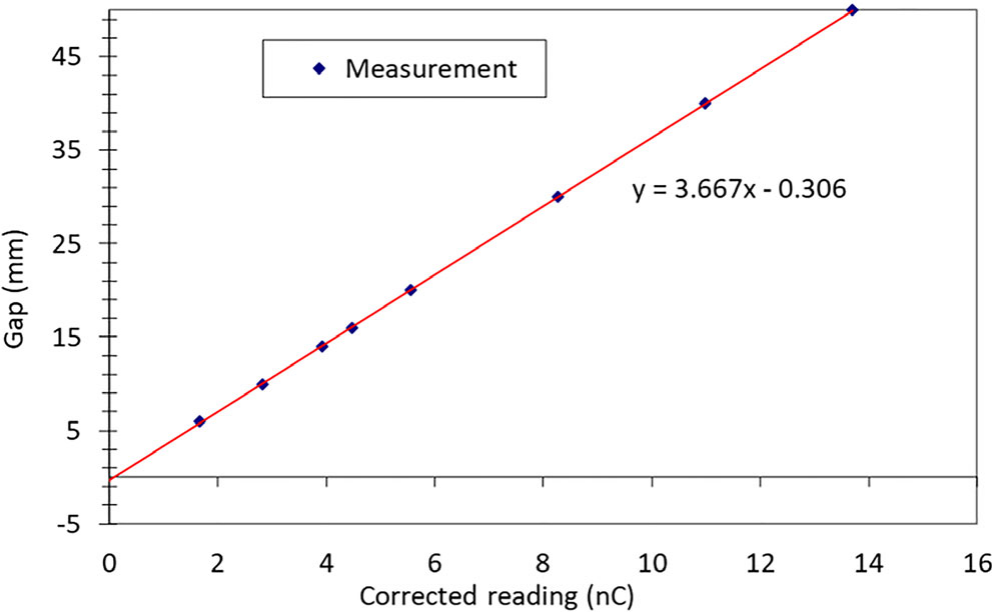
Sweeping‐gap‐based DLG measurement as modeling factor for MLC leaf end transmission. A linear fit of measured data, resulted in a −0.3 mm y intercept, which translates into a DLG of 0.3 mm for 6x photons. DLG, dosimetric leaf gaps; MLC, multileaf collimator.

The measured MLC leaf transmission factors were less than 0.5% for all energies, ranging from 0.3% to 0.5%. The values were in line with previously reported value^25^ and below the 2% threshold set in AAPM Task Group No. 50.^17^ Figure 4 displays the DLG measurement results for 6x photons using the sweeping‐gap technique. As the gap sizes were reduced, the corresponding corrected ionization chamber readings were recorded. The correction entails removal of MLC leaf transmission measured for closed leaves. A linear fit was subsequently performed to derive a theoretical negative gap which would reduce the ionization chamber reading to 0. The absolute value of the negative gap size is DLG, which quantifies the leaf end transmission with closed leaves. The sweeping‐gap‐based DLG measurements under a well‐controlled setting provided a coarse estimate of the optimal DLGs. The obtained values were subsequently fine‐tuned using the MatriXX QA and finalized using the film & IC QA. The results of DLG values are summarized in Table 2. The sweeping‐gap‐based DLG measurements provided a great starting point and also turned out to represent a close match to the final DLG values which greatly improved the speed and efficiency of the commissioning process.

**Table 2:** Evolution of the DLG values using the multilevel optimization scheme.

Energy	DLG measured by sweeping‐gap protocol (mm)	DLG fine‐tuned by MatriXX QA (mm)	DLG finalized by film & IC QA (mm)
6 MV	0.3	0.1	0.0
10 MV	0.4	0.5	0.5
15 MV	0.4	0.4	0.5
6xFFF	0.5	0.4	0.7
10xFFF	0.6	0.7	0.7

DLG, dosimetric leaf gaps.

### 3.4 Ionization chamber and film QA results

**Figure 5:**
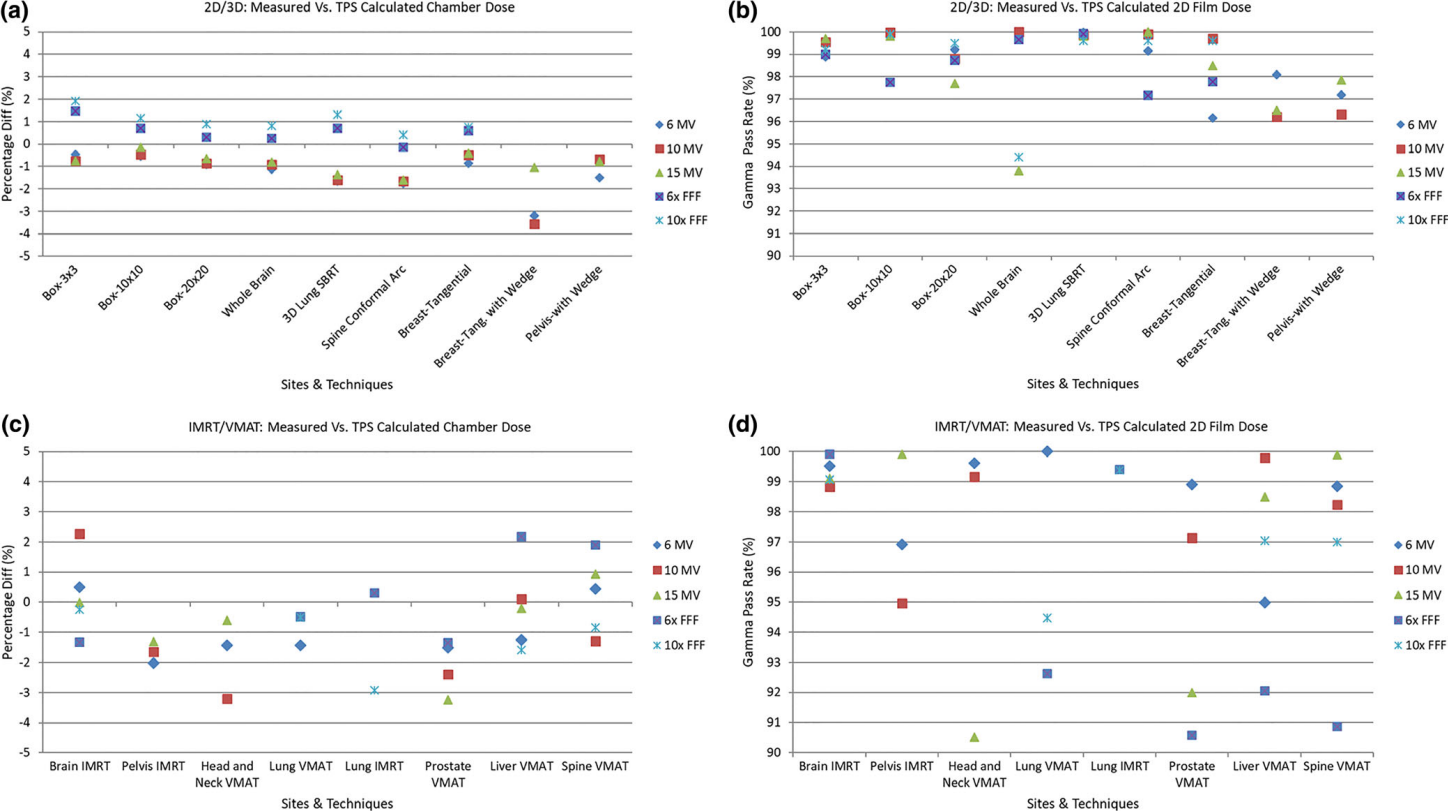
Comparison between calculated and measured doses, for different treatment sites, techniques and energies: (a). percent point dose differences of 2D/3D plans. The differences were calculated by subtracting TPS doses from ionization chamber measured doses, normalized by the TPS doses; (b). 2D film gamma pass rates of 2D/3D plans based on the 3%global/2 mm criteria; (c). percent point dose differences of IMRT/VMAT plans; and (d). 2D film gamma pass rates of IMRT/VMAT plans based on the 3%global/2 mm criteria. Note that not all energies were evaluated for several sites and techniques, since certain energies are not used for these sites and techniques in institution’s clinical practice. TPS, treatment planning system.

Figure 5 shows the point dose QA results for the commissioning plans using ionization chamber and film measurements. In summary, all point dose measurement results were within ±4%, and all film gamma pass rates were larger than 90%. For 2D/3D plans, the (mean ± SD) absolute point dose error was (1.3% ± 0.9%), and film gamma pass rate was (98.6% ± 1.6%). For IMRT/VMAT plans, the corresponding results were (1.0% ± 0.7%) and (97.0% ± 3.2%). For 2D/3D plans, all point dose measurements were within 2% agreement, except for the tangential breast plans with wedged fields. For the flattened beams (6x/10x/15x), the measured doses were consistently lower than the TPS computed doses. In contrast, for flattening‐filter‐free beams (6xFFF/10xFFF), a reverse trend was observed: the measured doses were consistently higher than the TPS calculated doses. For the IMRT/VMAT plans, there was no defined trend, though the measured doses were slightly lower than the TPS ones. In general, the 2D/3D plans are intrinsically without much modulation, which implies sensitivity to the daily machine output variations. On the other hand, the point doses of IMRT/VMAT plans are determined by complex mixture of factors including modulation level, DLG, output, and dose gradient across the chamber. As a result, the point dose errors had no distinct trends for IMRT/VMAT plans.

### 3.5 Mobius commissioning results

Mobius as an IMRT QA tool was initially commissioned only for the flattened 6x, 10x and 15x beams. The flattening‐filter‐free energies (6xFFF and 10xFFF) were deferred till later as enough clinical plans are accumulated for proper analysis. In interim, IC and film QA system is used for routine IMRT QA. For comparison, in Table 3, the optimized Mobius DLGs are listed along with the Eclipse DLG values for three flattened energies. Because of the differences in dose calculation algorithms (Collapsed cone convolution (CCC) vs AAA) and beam modeling, Mobius and Eclipse DLG values can be different, whereas Mobius DLGs can also have negative values, as shown in Table 3.

**Table 3:** Comparison of DLG values between Eclipse and Mobius.

Energy	Eclipse DLG (mm)	Mobius DLG (mm)
6 MV	0.0	‒1.0
10 MV	0.5	0.0
15 MV	0.5	‒2.25

DLG, dosimetric leaf gaps.

A glance at Table 4 data reveals that the point dose agreement between Eclipse calculations and ionization chamber and Mobius measurements were within ±5%. The corresponding 3D dose distributions evaluated with 3%global/3 mm gamma criteria resulted in larger than 90% passing rates. Note that the 3%/3mm criteria used for Mobius commissioning match our current clinical IMRT QA guidelines for 3D gamma analysis based on LINAC delivery logfiles. The average gamma passing rate encompassing all commissioning plans comparing Mobius and Eclipse dose maps was (97.0% ± 2.5%).

**Table 4:** The percent difference was calculated by subtracting the TPS dose from the IC dose, normalized by the TPS dose (column 1), or by subtracting the TPS dose from the Mobius dose, normalized by the TPS dose (column 2), or by subtracting the IC dose from the Mobius dose, normalized by the IC dose (column 3).

	Plans	IC vs. TPS (Point Dose)	Mobius vs. TPS (Point Dose)	Mobius vs. IC (Point Dose)	Mobius vs. TPS (3%global/3 mm 3D Gamma)
6 MV	Brain‐IMRT	0.51%	0.76%	0.25%	99.10%
	Pelvis‐IMRT	‒2.02%	‒0.16%	1.89%	99.90%
	HN‐VMAT	‒1.42%	1.84%	3.31%	97.70%
	Lung‐VMAT	‒1.42%	‒1.37%	0.06%	100.00%
	Prostate‐VMAT	‒1.51%	1.77%	3.33%	97.20%
	Liver‐VMAT	‒1.25%	‒1.30%	‒0.05%	98.60%
	Spine‐VMAT	0.45%	0.19%	‒0.26%	99.50%
10 MV	Brain‐IMRT	2.28%	‒0.12%	‒2.35%	95.00%
	Pelvis‐IMRT	‒1.64%	0.08%	1.75%	99.20%
	HN‐VMAT	‒3.21%	‒0.23%	3.08%	94.30%
	Prostate‐VMAT	‒2.39%	0.46%	2.92%	91.40%
	Liver‐VMAT	0.10%	‒3.11%	‒3.21%	97.30%
	Spine‐VMAT	‒1.30%	‒3.49%	‒2.22%	95.80%
15 MV	Brain‐IMRT	‒0.02%	1.89%	1.91%	93.90%
	Pelvis‐IMRT	‒1.31%	‒0.34%	0.98%	96.90%
	HN‐VMAT	‒0.61%	3.14%	3.77%	99.40%
	Prostate‐VMAT	‒3.24%	1.08%	4.46%	98.50%
	Liver‐VMAT	‒0.21%	‒3.84%	‒3.65%	92.80%
	Spine‐VMAT	0.94%	‒0.86%	‒1.79%	96.60%

TPS, treatment planning system.

## 4 DISCUSSION

The Eclipse model for Elekta LINACs with the Agility MLC head was not available until late 2015. Only limited number of cancer centers are using Eclipse to model Elekta LINACs. The predominant choice is Elekta’s own Monaco TPS or a third party TPS system including Pinnacle or Raystation. Considering recent noteworthy advancements as well as future Eclipse TPS developments, our institution decided to utilize Eclipse as a single TPS system to model both Varian and Elekta LINACs for external beam planning.

The AAPM recommendations^15^, ^16^ and vendor protocols were closely followed to complete commissioning tasks for modeling Elekta VersaHD LINACs using Varian Eclipse TPS. All Eclipse mandatory data for commissioning were methodically measured, including PDDs, beam inline, crossline and diagonal profiles, beam open and wedged field output factors, MLC leaf transmission, and dosimetric leaf gap values. Sun Nuclear Edge diode detector was used to measure PDDs and beam profiles at small fields, whereas Scanditronix CC13 ionization chamber was used for all other field sizes. The consistency of data was validated by cross‐checking both detector measurements at an intermediate 3 cm × 3 cm field with excellent agreement demonstrated in Fig. 1. The Eclipse‐modeled beams, as shown in Fig. 3, faithfully matched the measurements. The DLG values underwent fine‐tuning process utilizing MatriXX and IC and film measurements. As a final step Mobius was commissioned as an independent dose verification system utilized for routine clinical IMRT QA and additional model verification.

With optimized DLG values (Table 2), the IC absolute point dose variation was achieved within ±4% (Fig. 5), accompanied by larger than 90% gamma passing rates (3%global/2 mm) for all 2D film measurements. Our institution uses a nominal 90% gamma pass rate to determine plan pass/fail. The rationale for choosing 90% gamma passing rate as clinically acceptable was based on the TG119s average 88% gamma passing score for composite film measurements. Site‐specific tolerance limits and action limits, as recommended by TG218, have not been established at our institution yet and remain to be investigated in future. A 97.0% gamma passing rate for 3%global/3 mm criteria was obtained for comparison between Mobius and Eclipse 3D dose distributions (Table 4). It is worth pointing out that although the AAPM Task Group reports 119 and 218 recommend using the Van Dyk or global method for gamma dose distribution comparisons,^23^, ^26^ a very strong case can be made for using the local point method which is more stringent and reveals larger discrepancies relative to global approach.^27^

A recent publication on VersaHD model validation in Pinnacle reported >99.5% gamma pass rate for all plans with 3%global/3 mm criteria, and around 95% with 2%global/2mm criteria.^28^ Absolute percent point dose differences were <2% for all studied cases. Another study on VersaHD model validation in Monaco found an average gamma pass rate of (93.8% ± 4.7%) based on 2%global/2mm criteria for eight types of IMRT test field plans, and an average gamma pass rate of 95% based on 3%global/3 mm criteria for eight re‐planned patient cases.^2^ Absolute percent point dose differences measured in a homogeneous phantom were <2% for all cases in the same study. Based on 3%global/2mm gamma criteria, our average gamma pass rate for 71 plans was (97.9% ± 2.5%), which is comparable to the two aforementioned published studies. Including all 2D/3D and IMRT/VMAT plans, our model, on average, resulted in a (1.1% ± 0.8%) absolute percent point dose agreement between calculations and measurements. 9 of 71 plans had an absolute point dose error larger than 2%, with a maximum outlier of 3.6%. The larger errors could be caused by pinpoint chamber measurement uncertainties, setup uncertainties such as placing ionization chamber in sharp dose gradient regions, or by a complex plan utilizing excessive modulation, or ultimately by the limitations of Eclipse beam model accuracy. The ±4% result for VersaHD LINACs, however, was in contrast to a more stringent ±2% point dose accuracy achieved for two VitalBeam LINACs (Varian Medical Systems, Palo Alto, CA) commissioned at the same time at our institution. In essence, all attempts to further minimize dose variations and make improvements for a particular site or technique would negatively reflect on another site or technique by making repeated measurements worse. This implies that the optimized DLG values for VersaHD are not truly optimal, rather a compromise which reveals a suboptimal LINAC model. A common practical knowledge for matching calculated and measured dose distributions considers that as DLG increases the planned dose also increases and vice versa. This apparently does not hold for Elekta LINACs but is valid for Varian LINACs within Eclipse TPS. Thorough scrutiny of measured beam data and the corresponding modelling of mandatory fields in Eclipse showed an excellent agreement. Yet, when complex MLC movements and intensity modulations were introduced, Fig. 5, dosimetric discrepancies could not be minimized across the board by simply adjusting DLG values. It is intuitive to conjecture that it is caused by the modeling of Elekta machines in Eclipse. Varian and Elekta LINACs employ different MLC leaf designs, so DLG concept that works well for Varian machines may not be sufficient for Elekta LINACs. Third party TPS systems are modeling the MLC shape directly instead of the parameterization approach of Eclipse.^29^ Our previous commissioning and clinical experience with Pinnacle TPS did not reveal distinct measured dose differences related to either Varian or Elekta LINACs models.

Of note is that the Mobius vs. ionization chamber measurements were suboptimal for 15x. The current model represents a compromise after multiple trials with a realization that it is unachievable to simultaneously optimize both head and neck VMAT plans and liver VMAT plans. Optimizing one site will in turn make the other site worse, which is essentially echoing the scenario within Eclipse and indirectly revealing a modeling deficiency.

## 5 CONCLUSIONS

It is feasible to use Eclipse to model Elekta VersaHD LINACs for dose calculation and treatment planning. Nevertheless, future developments and major model improvements are still warranted for Elekta LINAC models within Eclipse platform to be on par with the corresponding Varian LINAC models.

## CONFLICT OF INTEREST

No conflict of interest.

## References

[1] Chang Z , Wu Q , Adamson J , et al. Commissioning and dosimetric characteristics of TrueBeam system: composite data of three TrueBeam machines. Med Phys. 2012;39:6981–7018. 23127092 10.1118/1.4762682

[2] Narayanasamy G , Saenz DL , Defoor D , Papanikolaou N , Stathakis S . Dosimetric validation of Monaco treatment planning system on an Elekta VersaHD linear accelerator. J Appl Clin Med Phys. 2017;18:123–129. 10.1002/acm2.12188 PMC5689924 28944979

[3] Tesfamicael B . Accuracy of dose calculation algorithms in Eclipse treatment planning system: an update. South Asian J Cancer. 2013;2:197. 10.4103/2278-330X.119892 PMC3889027 24455624

[4] Xia P , Murray E . 3D treatment planning system‐Pinnacle system. Med Dosim. 2018;43:118–128. 29580933 10.1016/j.meddos.2018.02.004

[5] Bodensteiner D . RayStation: external beam treatment planning system. Med Dosim. 2018;43:168–76. 29650302 10.1016/j.meddos.2018.02.013

[6] Olsen LA , Robinson CG , He GR , et al. Automated radiation therapy treatment plan workflow using a commercial application programming interface. Practical radiation oncology. 2014;4:358–367. 25407855 10.1016/j.prro.2013.11.007

[7] Davies GA , Clowes P , Bedford JL , et al. An experimental evaluation of the agility MLC for motion‐compensated VMAT delivery. Phys Med Biol. 2013;58:4643–4657. 23780400 10.1088/0031-9155/58/13/4643

[8] Vassiliev ON , Titt U , Ponisch F , et al. Dosimetric properties of photon beams from a flattening filter free clinical accelerator. Phys Med Biol. 2006;51:1907–1917. 16552113 10.1088/0031-9155/51/7/019

[9] Han Z , Ng SK , Bhagwat MS , Lyatskaya Y , Zygmanski P . Evaluation of MatriXX for IMRT and VMAT dose verifications in peripheral dose regions. Med Phys. 2010;37:3704–3714. 20831078 10.1118/1.3455707

[10] Childress N , Chen Q , Rong Y . Parallel/opposed: IMRT QA using treatment log files is superior to conventional measurement‐based method. J Appl Clin Med Phys. 2015;16:4–7. 10.1120/jacmp.v16i1.5385 PMC5689982 25679180

[11] Narayanasamy G , Saenz D , Cruz W , et al. Commissioning an Elekta Versa HD linear accelerator. J Appl Clin Med Phys. 2016;17:179–91. 10.1120/jacmp.v17i1.5799 PMC5690217 26894351

[12] Sievinen J , Ulmer W , Kaissl W . AAA photon dose calculation model in Eclipse. Palo Alto (CA): Varian Medical Systems. 2005;118:2894.

[13] Akino Y , Gibbons JP , Neck DW , Chu C , Das IJ . Intra‐and intervariability in beam data commissioning among water phantom scanning systems. J Appl Clin Med Phys. 2014;15:251–258. 10.1120/jacmp.v15i4.4850 PMC5875503 25207415

[14] Dieterich S , Sherouse GW . Experimental comparison of seven commercial dosimetry diodes for measurement of stereotactic radiosurgery cone factors. Med Phys. 2011;38:4166–4173. 21859018 10.1118/1.3592647

[15] Nath R , Biggs PJ , Bova FJ , et al. AAPM code of practice for radiotherapy accelerators: report of AAPM Radiation Therapy Task Group No. 45. Med Phys. 1994;21:1093–1121. 7968843 10.1118/1.597398

[16] Das IJ , Cheng CW , Watts RJ , et al. Accelerator beam data commissioning equipment and procedures: report of the TG‐106 of the Therapy Physics Committee of the AAPM. Med Phys. 2008;35:4186–4215. 18841871 10.1118/1.2969070

[17] Boyer A , Biggs P , Galvin J , et al. Basic applications of multileaf collimators. report of the aapm radiation therapy committee task group no. 50. Med Phys. 2001;72:16–40.

[18] Chang Z , Wang Z , Wu QJ , et al. Dosimetric characteristics of novalis Tx system with high definition multileaf collimator. Med Phys. 2008;35:4460–4463. 18975693 10.1118/1.2977668

[19] Dreindl R , Georg D , Stock M . Radiochromic film dosimetry: considerations on precision and accuracy for EBT2 and EBT3 type films. Z Med Phys. 2014;24:153–163. 24055395 10.1016/j.zemedi.2013.08.002

[20] Low DA , Harms WB , Mutic S , Purdy JA . A technique for the quantitative evaluation of dose distributions. Med Phys. 1998;25:656–661. 9608475 10.1118/1.598248

[21] Fraser D , Parker W , Seuntjens J . Characterization of cylindrical ionization chambers for patient specific IMRT QA. J Appl Clin Med Phys. 2009;10:2923. 19918222 10.1120/jacmp.v10i4.2923 PMC5720562

[22] Sanchez‐Doblado F , Hartmann GH , Pena J , et al. Uncertainty estimation in intensity‐modulated radiotherapy absolute dosimetry verification. Int J Radiat Oncol Biol Phys. 2007;68:301–310. 17448883 10.1016/j.ijrobp.2006.11.056

[23] Ezzell GA , Burmeister JW , Dogan N , et al. IMRT commissioning: multiple institution planning and dosimetry comparisons, a report from AAPM Task Group 119. Med Phys. 2009;36:5359–5373. 19994544 10.1118/1.3238104

[24] Kerns JR , Followill DS , Lowenstein J , et al. Reference dosimetry data and modeling challenges for Elekta accelerators based on IROC‐Houston site visit data. Med Phys. 2018;45:2337–2344. 29537634 10.1002/mp.12865 PMC6592280

[25] Bedford JL , Thomas MD , Smyth G . Beam modeling and VMAT performance with the agility 160‐leaf multileaf collimator. J Appl Clin Med Phys. 2013;14:4136. 23470941 10.1120/jacmp.v14i2.4136 PMC5714360

[26] Miften M , Olch A , Mihailidis D , et al. Tolerance limits and methodologies for IMRT measurement‐based verification QA: recommendations of AAPM Task Group No. 218. Med Phys. 2018;45:e53–e83. 29443390 10.1002/mp.12810

[27] Stojadinovic S , Ouyang L , Gu X , et al. Breaking bad IMRT QA practice. J Appl Clin Med Phys. 2015;16:5242. 26103484 10.1120/jacmp.v16i3.5242 PMC5690124

[28] Saenz DL , Narayanasamy G , Cruz W , Papanikolaou N , Stathakis S . Pinnacle3 modeling and end‐to‐end dosimetric testing of a Versa HD linear accelerator with the Agility head and flattening filter‐free modes. J Appl Clin Med Phys. 2016;17:192–206. 26894352 10.1120/jacmp.v17i1.5808 PMC5690210

[29] Ajo R Jr . A dosimetric evaluation of the Eclipse and Pinnacle treatment planning systems in treatment of vertebral bodies using IMRT and VMAT with modeled and commissioned flattening filter free (FFF) fields. 2016.

